# Early kinetics of serum Interleukine-17A and infarct size in patients with reperfused acute ST-elevated myocardial infarction

**DOI:** 10.1371/journal.pone.0188202

**Published:** 2017-11-22

**Authors:** Thomas Bochaton, Nathan Mewton, NDieme Thiam, Fabien Lavocat, Delphine Baetz, Nathalie Dufay, Cyril Prieur, Eric Bonnefoy-Cudraz, Pierre Miossec, Michel Ovize

**Affiliations:** 1 INSERM U1060, CarMeN laboratory, Université de Lyon, Groupement Hospitalier Est, Bron, France; 2 Unité de Soins Intensifs Cardiologiques, Hôpital Louis Pradel, Hospices Civils de Lyon, Lyon, France; 3 Service d’explorations fonctionnelles Cardiovasculaires, Hôpital Louis Pradel, Hospices Civils de Lyon, Lyon, France; 4 Centre d’investigation clinique de Lyon, Hôpital Louis Pradel, Hospices Civils de Lyon, Lyon, France; 5 Immunogénomique et Inflammation, Hôpital Edouard Herriot Pavillon P, Lyon, France; 6 NeuroBioTec, Groupement Hospitalier Est, Hôpital Neurologique Pierre Wertheimer, Lyon, France; Centre National de la Recherche Scientifique, FRANCE

## Abstract

**Background:**

Recently, it was shown that interleukin-17A (IL-17A) is involved in the pathophysiology of reperfusion injury and associated with infarct size (IS) in experimental models of myocardial infarction. Our aim was to evaluate whether the IL-17A serum level and the IL-17A active fraction was correlated with IS in humans.

**Methods:**

101 patients presenting with a ST-elevated Myocardial Infarction (STEMI) referred for primary percutaneous coronary intervention (PPCI) and 10 healthy controls were included. For each participant, blood samples at admission (H0) and 4 hours after admission (H4) were collected. IL-17A serum levels were assessed using ELISA and the active fraction was assessed with a functional test. IS was determined by peak troponin and peak CK levels for every patient and by contrast-enhanced cardiac magnetic resonance (ce-CMR) for 20 patients.

**Results:**

The IL-17A serum level was significantly increased in STEMI patients compared to healthy controls, (0.9 pg/mL IQR [0.0–3.2] at H0 and 1.0 pg/mL IQR [0.2–2.8] at H4 versus 0.2 pg/mL IQR [0.0–0.7] for healthy controls; p<0.005). At either time points, IL-17A levels did not correlate with IS as measured by peak troponin, peak CK pr ce-CMR. Also, no correlation was found between the active fraction of IL-17A and IS.

**Conclusion:**

Serum IL-17A level is significantly increased in patients at the early phase of acute MI compared to healthy controls. However, the level of IL-17A in the early hours after reperfusion does not correlate with IS.

## Introduction

Myocardial infarction (MI) is one of the main causes of death in the world [1]. Infarct size (IS) is associated with heart failure and mortality following MI [2,3]. Early reperfusion is currently the most effective treatment to reduce IS resulting from MI [4,5]. Although reperfusion reduces infarct size, it causes myocardial injury by itself [6]. This process is called ischemia-reperfusion (I/R) injury [7] and constitutes a target for the development of new therapies to reduce the final IS [8,9]. Among, the different mechanisms involved in I/R injury, inflammation appears to play a significant part in the final damage to the ischemic myocardium [10].

Three recovery phases have been described following myocardial infarction: the inflammatory phase, the proliferative phase and the maturation phase [11]. Inflammation has been implicated in the extension of reperfusion lesions, and in the pathogenesis of post-infarction remodeling [12]. Cardiomyocyte death after myocardial infarction triggers an intense inflammatory response. This response is mediated by endogenous signals known as Danger-Associated Molecular Patterns (DAMPs) that activate the innate immune system, via the Toll-Like Receptors (TLR) [13]. Activation of DAMPs-mediated signaling induces the production of chemokines and cytokines that trigger recruitment of leucocytes in the infarcted zone [14]. Tumor Necrosis Factor (TNF), Interleukin-1β (IL-1β) and Interleukin-6 (IL-6) are markedly increased during myocardial infarction [15,16]. These cytokines have multifunctional and pleiotropic effects. They can either be deleterious, protective or both [17–21].

Interleukin (IL)-17A belongs to the IL-17 family composed of 6 isoforms (IL-17A, IL-17B, IL-17C, IL-17D, IL-17E, and IL-17F) [22]. IL-17A is a pro-inflammatory cytokine involved in the pathogenesis of several inflammatory and auto-immune inflammatory diseases [23]. Recently, Liao *et al*. demonstrated in an experimental murine model that IL-17A contributed to I/R injury by increasing cardiomyocyte apoptosis and neutrophil infiltration [24]. They showed that IL-17A mRNA and protein levels in the myocardium significantly increased as early as first hours of reperfusion to reach a peak at 24h. They also demonstrated that inhibiting IL-17A *in vivo* significantly reduced the final infarct size and I/R injury.

In human patients, there is very little data on the association between IL-17A and IS. Cheng *et al*. showed that IL-17A was increased during myocardial infarction compared to stable angina or chest pain syndrome in a small selected population of patients [25]. Recently, Simon *et al*. showed that low serum levels of IL-17A were associated with a higher risk of major cardiovascular events in a cohort of patients with acute MI [26]. The role of IL-17A in human patients presenting with acute MI remains unclear and there is no data on IL-17A levels in the first hours following reperfusion and their relationship with IS.

Our main objective was therefore to assess IL-17A serum levels and IL-17A active fraction in the first hours of reperfusion and their relationship with other cytokines and IS in human patients presenting with acute ST-elevated MI (STEMI).

## Methods

### Study population

The study cohort was constituted by patients admitted to our institution, a tertiary referral university hospital, with a diagnosis of acute STEMI from 2012 to 2013. Our institutional review board and Ethics Committee (Comité d'éthique des Hospices Civils de Lyon, Professor Jean François Guérin) approved this retrospective monocentric study. All patients gave written informed consent.

STEMI was defined by the presence of clinical symptoms (chest pain) associated with an ST elevation of more than 2 mm in two contiguous leads on a standard 12-lead electrocardiogram, and significant troponin-I elevation according to the European Society of Cardiology [27]. All patients admitted to our institution underwent a coronary angiogram at the admission with reperfusion by primary percutaneous intervention (PCI).

101 patients were included in our study. They had complete myocardial enzyme release assessment and/or contrast enhanced Cardiac Magnetic Resonance (ce-CMR) assessment within the first month following admission. 20 patients from this cohort underwent a ce-CMR study to assess myocardial IS.

Patients with a medical history of auto-immune disease or active inflammatory disease were excluded from the study.

### Blood sampling protocol

**Fig 1:**
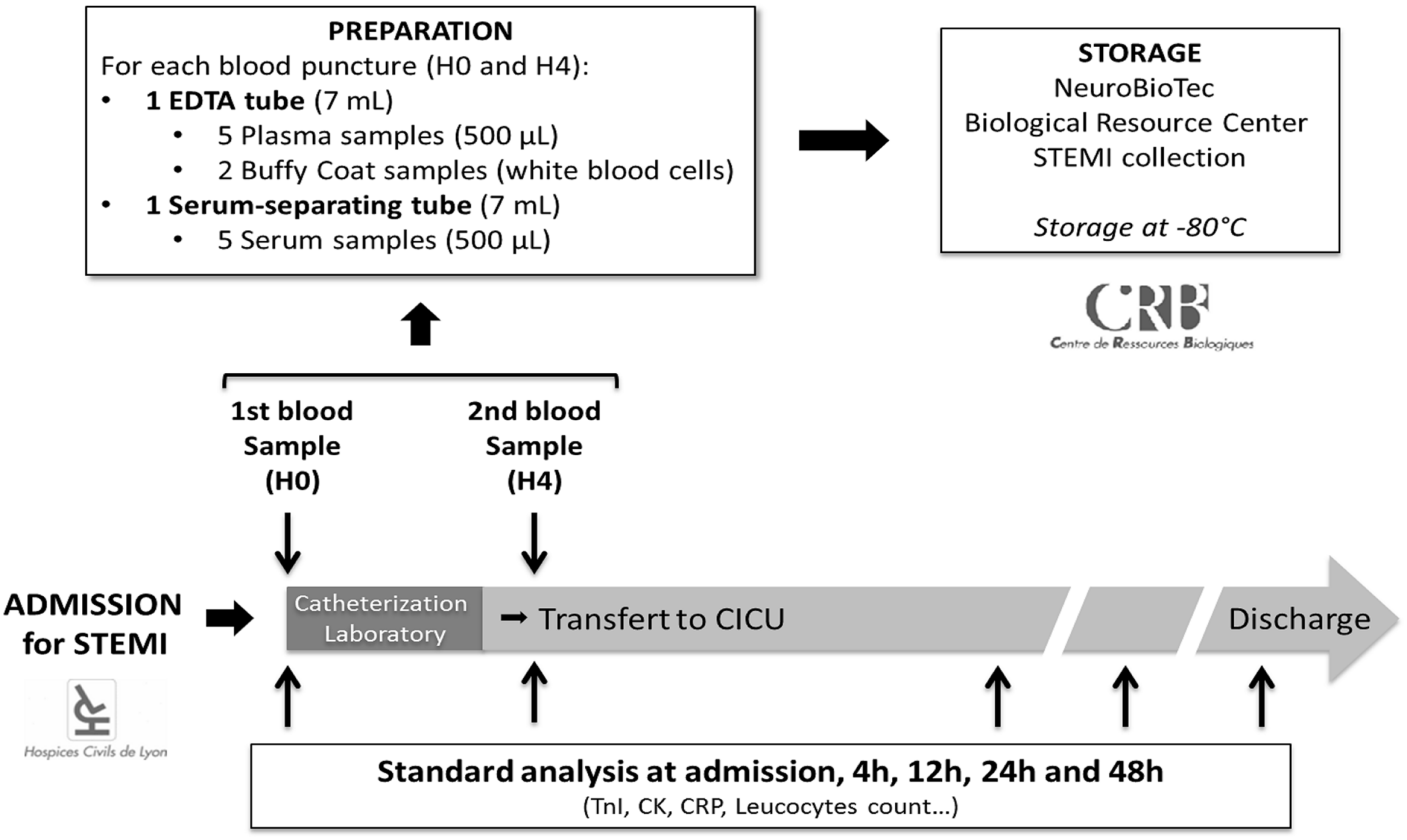
Blood samples collection protocol for STEMI cohort at our institution.

All sera from our study population were stored at the hospital biobank (NeuroBioTec Biological Resource Center). Two blood samples were collected for each patient. One sample was collected upon admission in the catheterization laboratory (H0) and one sample was collected four hours after admission (H4), in the Cardiac Intensive Care Unit (CICU). Sera were prepared and stored at -80°C as presented in Fig 1. Upon extraction, all samples from our study population were thawed only once to avoid cytokine alteration.

Ten sera samples from ten healthy volunteers were also obtained to assess the basal level of cytokines in a reference population.

### Cytokine measurement ELISA assay

The known normal values of IL-17A vary according to studies and assays used. It usually ranges from 2 pg/mL to 25 pg/mL [28–30].

IL-17A concentrations were measured using the Human IL-17A High Sensitivity ELISA kit (Affymetrix eBioscience, San Diego CA, USA). For IL-6 we used the Human IL-6 Quantikine ELISA Kit (R&D Systems, Minneapolis MN, USA). IL-8 concentrations were assessed with the Human CXCL8/IL-8 Quantikine ELISA Kit (R&D Systems, Minneapolis, MN). IL-1β concentrations were measured with the Human IL-1 beta ELISA Ready-SET-Go (Affymetrix eBioscience, San Diego CA, USA). The limit of detection for IL-17A was 0.5 pg/mL, 0.7 pg/mL for IL-6, 7.5 pg/mL for IL-8 and 2 pg/mL for IL-1β.

For IL-17A, patients with serum level below the test sensibility (0.5 pg/mL) were considered as seronegative and patients with serum level above the test sensibility were considered as seropositive for IL-17A.

### Functional test

In addition to the ELISA assay, an “in-house” functional test was performed for 20 patients in which IS was also assessed by CMR. Human Umbilical Vein Endothelial Cells (HUVEC) were collected from human umbilical cords (collagenase perfusion). HUVEC were cultured in EGM^™^-2 medium (Lonza, Basel, Switzerland) with Fetal Bovine Serum (FBS) and incubated overnight at 37°C. Sera from patients were incubated at 56°C for 30 min in order to inactivate complement.

HUVEC were then exposed to 10% of patients’ sera (diluted in EGM^™^-2 medium) with or without addition of anti-IL-17A antibody at the concentration of 10 μg/mL (R&D Systems, Minneapolis MN, USA). After 48h of incubation, supernatants were collected and IL-8 levels were measured by ELISA (R&D Systems, Minneapolis MN, USA). For each patient, the IL-8 delta (ΔIL-8) corresponding to the difference between IL-8 produced by HUVECs treated with patients’ serum alone and HUVECs treated with patient serum and anti-IL-17A antibody was calculated. The ΔIL-8 corresponds to HUVECs IL-8 secretion induced by IL-17A (= active fraction of IL-17A). All experiments were duplicated. This test was previously described and validated [31].

### Other biomarkers measurements

Blood samples for serum troponin I (Immunoassay Access^®^ AccuTnI^™^ Troponin I Assay) and creatine kinase levels (Beckman Coulter Inc, expressed in IU/L) were taken at admission, at 4 hours, 12 hours, 24 hours and 48 hours after admission.

C-reactive protein (CRP), leucocytes and neutrophils count and creatinine serum levels were also measured upon admission. Glomerular Filtration Rate was estimated using the Modification of Diet in Renal Disease formula (MDRD).

### Clinical data

Cardiovascular Disease (CVD) Risk Factors, treatment at admission, in-hospital management were collected for each patient. Ischemia time was defined as the time from symptom onset to complete reperfusion after percutaneous coronary angioplasty. Left Ventricular Ejection Fraction (LVEF) was evaluated using the Biplan method by routine cardiac ultrasound during the ICU admission.

### Cardiac magnetic resonance imaging protocol

20 of the 101 patients underwent a ce-CMR study one month after initial admission for MI. Patients were scanned in a supine position on a 1.5 T MAGNETOM Avanto TIM system (Siemens, Erlangen, Germany).

Ce-CMR were analysed as previously described [32–34]. Briefly, LV function at rest was assessed with retrospective ECG-gated steady-state free precession pulse cine sequences (cine TrueFISP) in long and short axis views in the true heart axis.

An intravenous bolus of Gadolinium-DOTA at a dose of 0.2 mmol/kg body weight for the late enhancement analysis was performed. Late gadolinium enhancement (LGE) was evaluated 10 minutes after contrast injection using a 3D-gradient spoiled TurboFLASH sequence with a selective 180° inversion recovery pre-pulse, in the short axis covering the whole ventricle.

MI was identified by LGE within the myocardium, defined quantitatively by myocardial postcontrast signal intensity > 2SD above that within a reference of region remote non-infarcted myocardium within the same slice with the post-processing software Osirix (OsiriX Foundation, Geneva, Switzerland).

### Statistical analysis

Data are expressed as mean ± standard deviation (SD) or median and interquartile range depending on their distribution. The majority of the biochemical parameters were characterized by their high non-Gaussian distribution, using the Shapiro-Wilk test. Comparisons between groups were performed using unpaired *t* test for continuous variables and Pearson χ^2^ test or Fisher’s exact test for categorical variables. For Gaussian distributed values, differences among groups were assessed using one-way ANOVA followed by the Dunnett test. For non-Gaussian distributed values difference among groups was tested using the non-parametric Kruskal-Wallis test followed by the Dunn’s test for multiple comparisons. Correlations were tested by Spearman correlation for non-Gaussian distribution values and Pearson correlation for Gaussian distributed values. A two-tailed p-value <0.05 was considered as significant. All statistical analyses were performed with GraphPad 7.01 (Prism).

## Results

### Baseline characteristics

Baseline demographic and clinical characteristics of the study population are presented in Table 1. Briefly, patient age was 61±11 years, 78% males with an ischemia time of 180 min; interquartile range IQR [120–270 min]. The left anterior descending (LAD) coronary artery was the culprit coronary in 40.6% cases and the left ventricular ejection fraction was 45% IQR [40–55].

**Table 1:** Baseline characteristics of the study population.

Demographic Characteristics and Clinical Presentation	(n = 101)
Age (yr)*	61±11
Sex (M/F)	79/22
Hypertension (no.)	42
Smoking (no.)	41
Dyslipidemia (no.)	40
Diabetes (no.)	14
Prior history of coronary artery disease (no.)	16
Ischemia time (min)^†^	180 [120–270]
Killip at admission	
Killip 1	90
Killip ≥ 2	11
Infarct-related artery (no.)	
Left anterior descending coronary artery	41
Right coronary artery	41
Left circumflex coronary artery	18
Left main coronary artery	1
Left ventricular ejection fraction (%)^†^	45 [40–55]

*Mean±SD.

^†^Median value and interquartile range.

The principal biological and in-hospital management characteristics are presented in Tables 2 and 3 respectively.

**Table 2:** Principal biological characteristics of the study population at admission and myocardial biomarkers peak values.

Biological Characteristics*	(n = 101)
Serum creatinine (μmol/L)	77 [64–96]
MDRD (mL/min/1.73m^2^)^†^	88 ± 32
CRP (mg/L)	3.2 [1.9–6.7]
Leucocytes (G/L)	12.0 [9.4–15.2]
Neutrophil granulocytes (G/L)	9.3 [7.3–11.9]
Lymphocytes (G/L)	1.7 [1.4–2.3]
Peak troponin level (μg/L)	98.2 [57.6–167.5]
Peak CK level (IU/L)	2748 [1717–4021]

*Median value and interquartile range.

^†^Mean±SD. MDRD: Modification of Diet in Renal Disease formula; CRP: C-Reactive Protein; CK: Creatine Kinase

**Table 3:** In-hospital management.

Characteristics	(n = 101)
PCI (no.)	97 (96.0%)
Thrombolysis (no.)	6 (5.9%)
Coronary artery bypass surgery (no.)	0 (0%)
Antiplatelet therapy (at discharge)	
Aspirin (no.)	97 (96.0%)
P2Y12 inhibitor (no.)	96 (95.0%)
Low molecular weight heparin (no.)	95 (94.1%)
TIMI flow pre-PCI (no.)	
TIMI 0	72 (71.3%)
TIMI ≥ 1	29 (28.7%)
TIMI flow post-PCI (no.)	
TIMI ≤ 2	11 (10.9%)
TIMI 3	90 (89.1%)

PCI: percutaneous coronary angioplasty; TIMI: Thrombolysis in Myocardial Infarction.

Healthy controls were 53.3±4.7 years old. There were five males and five females. They had no known previous history of Cardiovascular Disease (CVD) and no Risk Factors.

### IL-17A measurements

**Fig 2:**
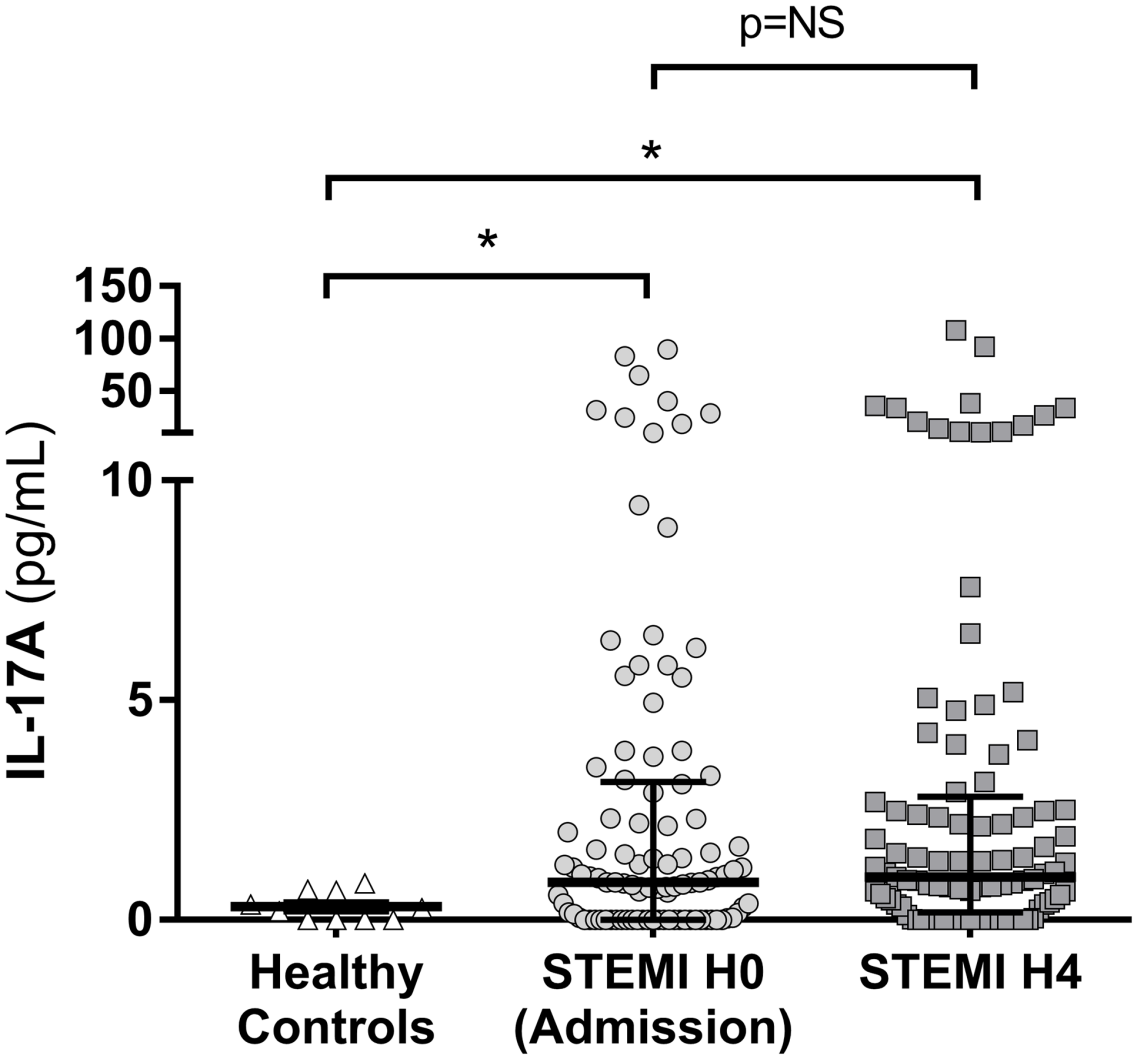
IL-17A level in STEMI patient at admission (H0) and 4 hours after admission (H4). IL-17A was measured by ELISA at H0 and H4 in patients with acute myocardial infarction (MI). IL-17A was increased as early as the first hours of acute MI, with a median value of 0.9 pg/mL IQR [0.0–3.2] at H0 and 1.0 pg/mL IQR [0.2–2.8] at H4 compared to 0.2 pg/mL IQR [0.0–0.7] for healthy controls. STEMI: ST-Segment Elevation Myocardial Infarction. NS: not significant, *p<0.05.

IL-17A was significantly increased at admission (H0) in the serum of the STEMI patients (0.9 pg/mL IQR [0.0–3.2]) compared to 0.2 pg/mL IQR [0.0–0.7] for healthy controls (p = 0.032). Four hours after admission (H4), IL-17A median level remained significantly increased at 1.0 pg/mL (0.2–2.8) compared to healthy controls (p = 0.015). There was no significant variation of IL17A levels between H0 and H4 (p>0.999) (Fig 2).

34 STEMI patients (33.7%) were seronegative (IL-17A < 0.5 pg/mL) for IL-17A at H0 and 30 patients (29.7%) remained seronegative at H4.

**Fig 3:**
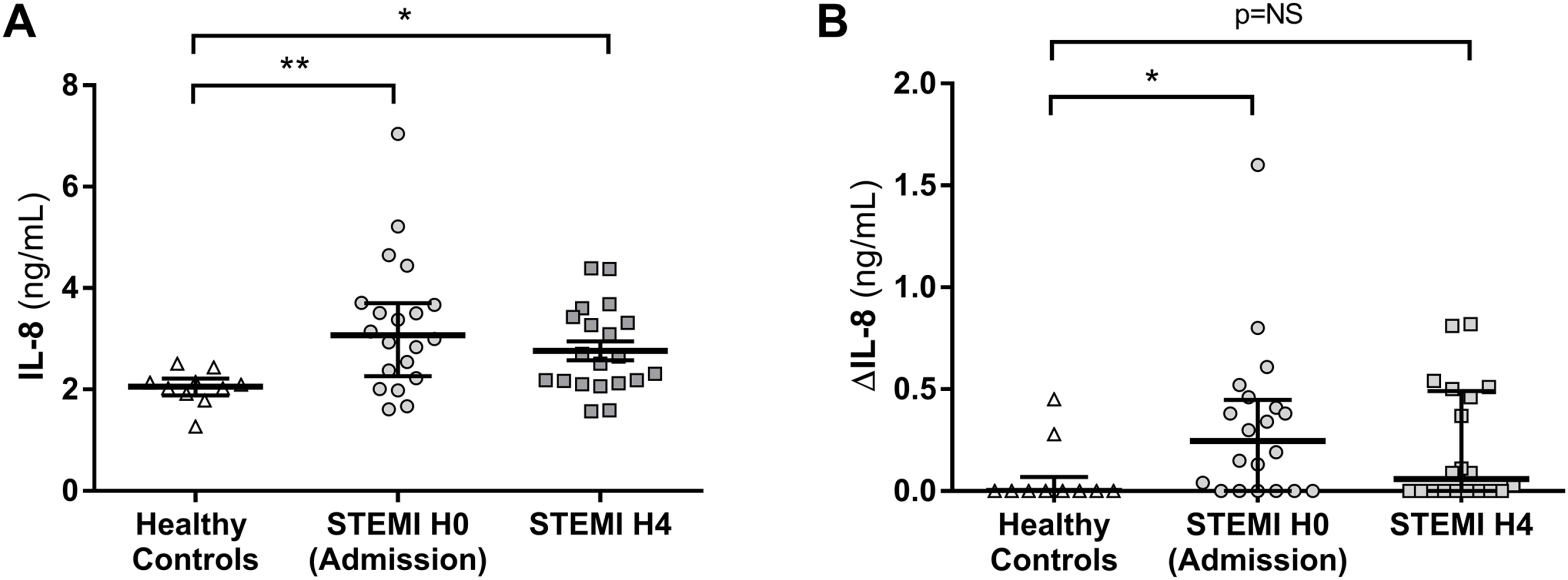
IL-17A functional test with Human Umbilical Vein Endothelial Cells (HUVEC). **A**; HUVECs were incubated 48 hours with the serum of each patient. IL-8 production by HUVECs was significantly higher in STEMI patient (at H0 and at H4) compared to healthy control. **B**; HUVECs were also incubated with each patient serum in the presence of anti-IL-17A antibody (neutralizing antibody). The difference between IL-8 secretion by HUVECs without and with IL-17A neutralizing antibody (named ΔIL-8) represented the secretion of IL-8 due to IL-17A. ΔIL-8 was significantly increased at H0 for STEMI patients compare to healthy controls but not at H4. STEMI: ST-Segment Elevation Myocardial Infarction. *p<0.05, **p<0.01.

As shown on Fig 3A, HUVECs produced significantly more IL-8 when incubated with STEMI patient serum than with healthy control serum (n = 20 patients for the STEMI group and n = 10 patients for the control group). The median value was 3.07 ng/mL IQR [2.26–3.70] at H0 and 2.58 IQR [2.13–3.40] at H4 for STEMI patients versus 2.06 ng/mL IQR [1.88–2.22] for healthy controls (p = 0.003 and p = 0.04 respectively). After incubation of HUVECs with each patient serum in the presence of anti-IL-17A antibody (neutralizing antibody) we assessed the specific IL-17A induction of IL-8 production. The production of IL-8 by HUVECs was significantly decreased because of the neutralization of IL-17A. As shown on Fig 3B, ΔIL-8 was significantly increased for STEMI patients at H0 with a median value of 0.25 ng/mL IQR [0.0–0.45] compared to 0.0 ng/mL IQR [0.0–0.07] for healthy controls (p = 0.03). At H4, ΔIL-8 decreased with a median value of 0.06 ng/mL IQR [0.0–0.49], comparable to healthy controls (p = NS compared to healthy controls).

### IL-17A relationship with IS

There was no correlation between IL-17A levels (n = 101 patients) at H0 or H4 and troponin peak in STEMI patients (r = -0.025, p = 0.80 and r = 0.044, p = 0.66 respectively). There was no correlation between IL-17A levels and CK peak at H0 and H4 (r = -0.034, p = 0.74 at H0 and r = 0.023, p = 0.82 at H4). Finally, there was no correlation between IL-17A levels at H0 and H4 and IS by CMR (r = -0.26, p = 0.28 and r = -0.29, p = 0.23 respectively).

**Fig 4:**
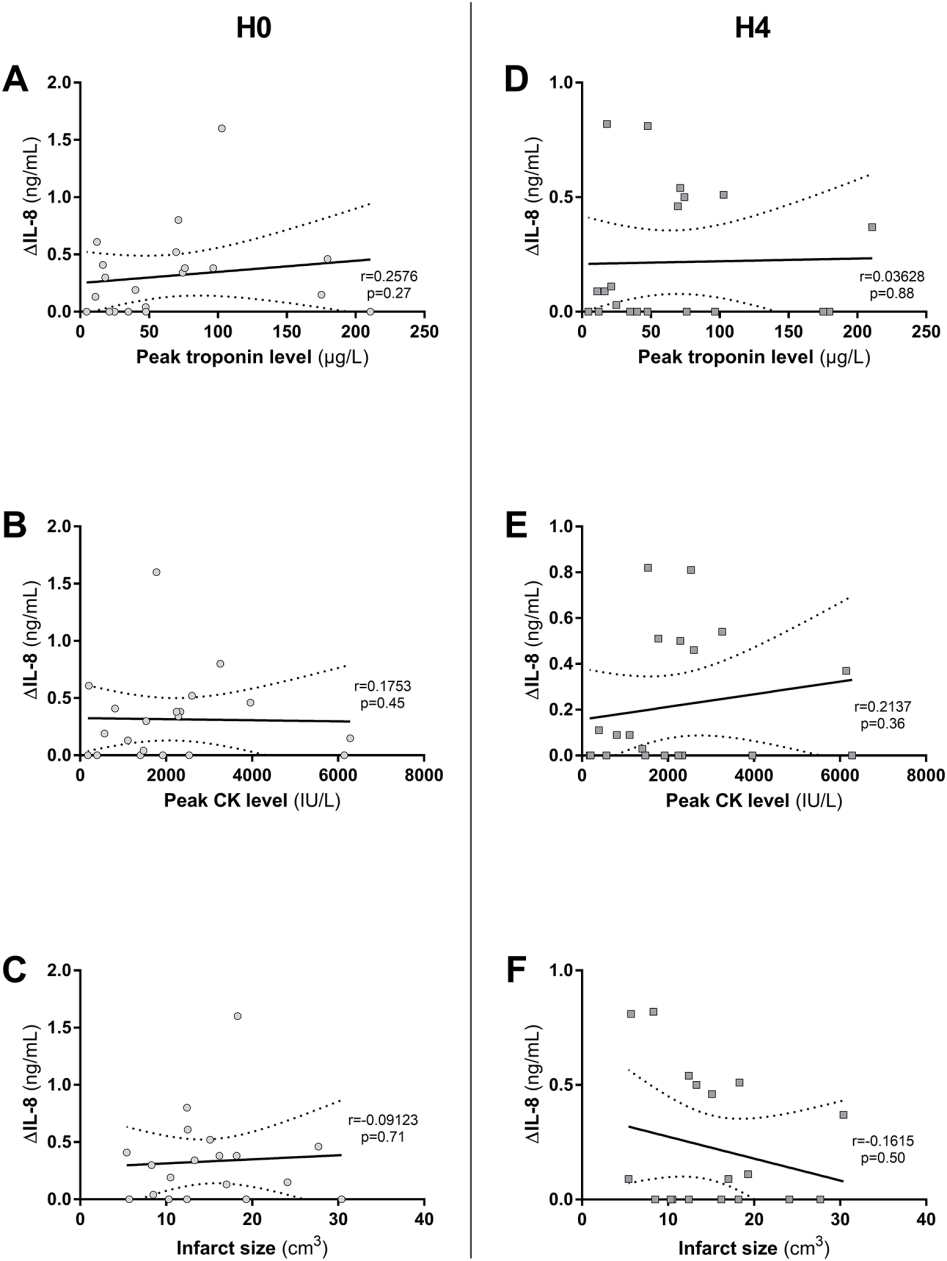
IL-17A activity assessed by ΔIL-8 on Human Umbilical Vein Endothelial Cells (HUVEC) and its correlation with infarct size (IS) in STEMI patients. **A, B and C**; IL-17A activity (ΔIL-8) at H0 was not correlated with IS as measured by peak troponin level (A) (r = 0.2576, p = 0.27) peak CK level (B) (r = 0.1753, p = 0.45) or Cardiac Magnetic Resonance (CMR) (C) (r = -0.09123, p = 0.71). **D, E and F**; ΔIL-8 at H4 was not correlated with IS as measured by peak troponin level (D) (r = 0.03628, p = 0.88) peak CK level (E) (r = 0.2137, p = 0.36) or CMR (F) (r = -0.1615, p = 0.50). Correlations were tested using Spearman correlation. CK: Creatine Kinase.

No significant correlation was found between ΔIL-8 (n = 20 patients) and troponin peak at H0 and H4 (r = 0.26, p = 0.27 and r = 0.036 and p = 0.88 respectively). In the same way there was no correlation between ΔIL-8 and CK peak at H0 and H4 (r = 0.18, p = 0.45 and r = 0.26, p = 0.27 respectively). The same results were found with infarct size assessed by CMR and ΔIL-8 at H0 and H4 (r = 0.09, p = 0.71 and r = 0.16, p = 0.50 respectively) (Fig 4).

### Other inflammatory markers in STEMI patients

IL-6 was 2.1 pg/mL IQR [0.0–4.2] at H0 and 4.7 pg/mL IQR [2.0–11.0] at H4. No correlation was found between IL-6 level at H0 and peak troponin or peak CK levels in our population (r = 0.09, p = 0.36 and r = -0.07, p = 0.46 respectively). Conversely, at H4 IL-6 levels were significantly correlated with IS as measured by peak troponin and CK level (respectively r = 0.38, p<0.0001 and r = 0.22, p = 0.029) (n = 101 patients).

There was no significant increase in IL-1β at H0 or H4 in our study population compared to healthy volunteers. Median serum level was 0.30 pg/mL IQR [0.16–1.53] at H0 and 0.67 pg/mL IQR [0.44–4.11] at H4 (p = 0.19 H0 versus H4) (n = 101 patients).

**Fig 5:**
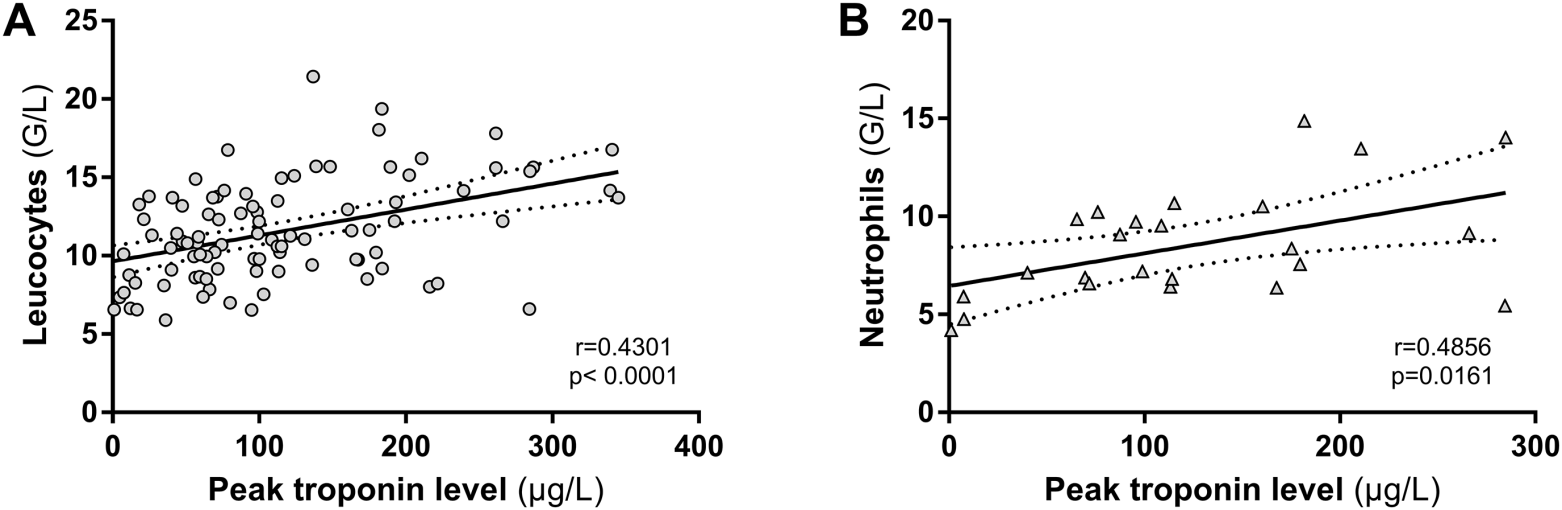
Leucocytes and neutrophil granulocytes at 24 hours (H24) and their correlation with myocardial infarct size (IS). **A**; Leucocytes count at H24 was significantly correlated with IS as measured by peak troponin release. **B**; At H24 neutrophil count was available for 24 patients only. For these patients, we found a significant correlation between neutrophil count and IS as measured by peak troponin release. Dotted line shows 95% confidence bands.

A significant correlation was found between leucocyte count at H24 and infarct size assessed by peak troponin release (r = 0.43, p<0.0001) (n = 95 patients) (Fig 5A). We also found a significant correlation between neutrophil count at H24 and myocardial IS assessed by peak troponin release (r = 0.49, p = 0.02) (Fig 5B) (n = 24 patients).

## Discussion

Our study has four main findings: 1) IL-17A prior to reperfusion and in the first hours following reperfusion was significantly increased at the acute phase of myocardial infarction when compared to healthy controls. However, one third of STEMI patients were seronegative for IL-17A in the first hours following reperfusion; 2) early IL-17A levels were not correlated with markers of IS; 3) Serum from STEMI patients stimulates HUVEC through the secretion of IL-8 which is a powerful neutrophils chemoattractant. 4) IL-6 and CRP were significantly increased at the acute phase of MI and IL-6 was significantly correlated with IS.

### IL-17A level after MI

Few studies have studied IL-17A level during MI in human and their results remain controversial. In a report by Hashmi *et al*. [35]. realized in patients with stable and unstable coronary artery disease, IL-17A was significantly increased in patient with MI. Patients had much higher IL-17A level than our population (mean at 307.42 pg/mL versus median at 1.0 pg/mL for our population). This important difference may be due to the fact that blood sampling in Hashmi study was done within the first 48h, while in our study, blood samples were collected significantly earlier, at admission and 4h after admission. IL-17A may increase during the first 48h hours following MI. The inflammatory response to ischemic myocardial infarction develops in the following 24 hours after reperfusion. Its peak is not well known, and might be situated at 24 hours as suggested by Liebetrau *et al*. [36]. In an experimental murine model of MI, it was shown that IL-17A messenger ribonucleic acid (mRNA) rose progressively until 24h and then began to decrease until 72h after ischemia reperfusion [24].

The level of IL-17A in our study was very low with a median of 1.0 pg/mL and 29.7% of IL-17A seronegative patients at the acute phase of STEMI. However, these results are close to those that have been published by Eid *et al*. and Simon *et al*. in recent studies, in MI patients [26,37]. Furthermore, the values found after MI seem to be lower than values reported in rheumatoid arthritis, with a mean level of 11.25 pg/mL [38].

Previous clinical studies have evaluated the level of IL-17A during MI [25,35,37] or the relationship between the level of IL-17A and cardiovascular outcome [26], but none had evaluated the relationship between IL-17A level and IS in human. Our study is the first to assess this relationship. No correlation was found at the early hours following reperfusion of MI (at H0 and H4) between IL-17A level and IS as measured by troponin and creatine kinase (CK) release. These results suggest that IL-17A is slightly activated in the initial hours following MI, but might significantly increase at a later stage. It has been shown that IL-17A increases during the 72 hours following MI in mice [24]. IL-17A could therefore be implicated in a later phase of inflammation, more than in the first hours following reperfusion in human patients.

Furthermore, our study is the first to evaluate the capacity of STEMI patient’s serum to induce IL-8 by HUVEC. Indeed, patient’s serum at the acute phase of STEMI, as early as admission, contains cytokines and other molecules that are able to induce IL-8 excretion by endothelial cells (HUVEC) when compared to serum from healthy volunteers. This result suggests a significant inflammation activation in first hours of STEMI that is able to induce IL-8 secretion by endothelial cells. Interestingly IL-8 is a powerful chemokine involved in neutrophils recruitment in the injured area [39,40]. It has been shown that neutrophils are the first immune cells recruited in the infarcted area [41]. IL-17A could participate in this recruitment via the activation of endothelial cells.

### Inflammation and MI

Conversely to IL-17A, other inflammatory markers such as IL-6, CRP, leucocytes and neutrophil granulocytes at the same stage and at 24 hours were correlated with IS. These results confirm data presented in previous studies and represent a quality marker for our study. IL-6 has been reported to be increased during myocardial infarction, but its correlation with IS remains controversial [42,43]. In our study we found that IL-6 was significantly correlated with IS as assessed by peak troponin and CK levels. Prior reports have shown that CRP gradually increased during the first 48h after myocardial infarction [42,44]. We also confirmed the previous data published by Chia *et al*. showing the increase of leucocyte and neutrophil counts and their significant correlation with IS [45]. Taken together, these results support the fact that inflammation is an important factor at the acute phase of MI in the following 48 hours after reperfusion.

Inflammation is currently an orphan therapeutic target after myocardial infarction. Our data show the complex and intense inflammatory response in the myocardial tissue after infarction. Further studies are warranted to better understand the complex kinetics and interaction between inflammatory cells, injured tissue and cytokines that result in additional damage to the myocardium. This is needed in order to find potential efficient therapeutic targets that could efficiently reduce infarct size in the early days after reperfusion and explore anti-inflammatory drugs in this setting. Recently, Deftereos *et al*. showed in a pilot study that colchicine (that has anti-inflammatory effects) significantly reduces IS in patients treated with Colchicine for 5 days, compared to patients treated with placebo [46]. Further experimental and clinical trials are needed to assess the effect of potent anti-inflammatory drugs on infarct size and clinical outcomes, but also on cytokine and inflammatory marker kinetics.

## Limits of our study

Our study was monocentric and retrospective and this induces a potential risk for selection bias. Further, blood samples were collected at the early phase of acute MI (H0 and H4), and we did not assess IL-17A after H4. As shown in an experimental study on mice, IL-17A RNA levels peaked at 24 hours after MI [24]. Our data only allows us to assess the early phase of reperfusion and therefore is incomplete. This also raises the limitation of direct measurement of cytokine levels in the myocardial tissue and their correlation with serum levels. In our study we did not perform myocardial biopsies, that could have shown different results in terms of IL17A expression. Finally, we had a small sample size for the comparison with CMR IS.

## Conclusion

IL-17A is significantly higher in the serum of patients at the early phase of acute MI following reperfusion compared to healthy controls. Early IL-17A levels and active fraction of IL-17A at the acute phase of MI did not correlate with IS. However, serum of STEMI patients induce the secretion of the powerful chemoattractant IL-8 by HUVECs.

## References

[1] World Health Organization. Global Atlas on cardiovascular disease prevention and control. 2011;

[2] Burns RJ, Gibbons RJ, Yi Q, Roberts RS, Miller TD, Schaer GL, et al The relationships of left ventricular ejection fraction, end-systolic volume index and infarct size to six-month mortality after hospital discharge following myocardial infarction treated by thrombolysis. J Am Coll Cardiol. 2002;39: 30–36. doi: 10.1016/S0735-1097(01)01711-9 11755283 10.1016/s0735-1097(01)01711-9

[3] Herlitz J, Karlson BW, Hjalmarson A. Ten year mortality in relation to original size of myocardial infarct: results from the Gothenburg metoprolol study. Heart. 1994;71: 238–241. doi: 10.1136/hrt.71.3.238 10.1136/hrt.71.3.238 PMC483660 8142192

[4] Marioko P, Libby P, Cinks W, Boioo CNI, Shiel W, Sober BE. Coronary Artery Reperfusion and the extent of myocardial necrosis. J Clin Invest. 1972;51. 10.1172/JCI107090 PMC332971 5056663

[5] Ginks WR, Sybers HD, Maroko PR, Covell JW, Sobel BE RJJ. Coronary artery reperfusion. II. Reduction of myocardial infarct size at 1 week after the coronary occlusion. J Clin Invest. 1972;51: 2717–2723. doi: 10.1172/JCI107091 5056664 10.1172/JCI107091 PMC332972

[6] Piper HM, García-Dorado D, Ovize M. A fresh look at reperfusion injury. Cardiovascular Research. 1998 pp. 291–300. doi: 10.1016/S0008-6363(98)00033-9 9709390 10.1016/s0008-6363(98)00033-9

[7] Yellon DM, Hausenloy DJ. Myocardial reperfusion injury. N Engl J Med. 2007;357: 1121–1135. doi: 10.1056/NEJMra071667 17855673 10.1056/NEJMra071667

[8] Ovize M, Baxter GF, Di Lisa F, Ferdinandy P, Garcia-Dorado D, Hausenloy DJ, et al Postconditioning and protection from reperfusion injury: where do we stand? Position paper from the Working Group of Cellular Biology of the Heart of the European Society of Cardiology. Cardiovasc Res. 2010;87: 406–23. doi: 10.1093/cvr/cvq129 20448097 10.1093/cvr/cvq129

[9] Piot C, Croisille P, Staat P, Thibault H, Rioufol G, Mewton N, et al Effect of cyclosporine on reperfusion injury in acute myocardial infarction. N Engl J Med. 2008;359: 473–81. doi: 10.1056/NEJMoa071142 18669426 10.1056/NEJMoa071142

[10] Marchant DJ, Boyd JH, Lin DC, Granville DJ, Garmaroudi FS, McManus BM. Inflammation in myocardial diseases. Circ Res. 2012;110: 126–44. doi: 10.1161/CIRCRESAHA.111.243170 22223210 10.1161/CIRCRESAHA.111.243170

[11] Frangogiannis NG. Regulation of the inflammatory response in cardiac repair. Circ Res. 2012;110: 159–73. doi: 10.1161/CIRCRESAHA.111.243162 22223212 10.1161/CIRCRESAHA.111.243162 PMC3690135

[12] Frangogiannis NG. The inflammatory response in myocardial injury, repair, and remodelling. Nat Rev Cardiol. Nature Publishing Group; 2014;11: 255–65. doi: 10.1038/nrcardio.2014.28 24663091 10.1038/nrcardio.2014.28 PMC4407144

[13] Timmers L, Pasterkamp G, de Hoog VC, Arslan F, Appelman Y, de Kleijn DP V. The innate immune response in reperfused myocardium. Cardiovasc Res. 2012;94: 276–83. doi: 10.1093/cvr/cvs018 22266751 10.1093/cvr/cvs018

[14] Frangogiannis NG. The immune system and cardiac repair. Pharmacol Res. 2008;58: 88–111. doi: 10.1016/j.phrs.2008.06.007 18620057 10.1016/j.phrs.2008.06.007 PMC2642482

[15] Herskowitz A, Choi S, Ansari AA, Wesselingh S. Cytokine mRNA expression in postischemic/reperfused myocardium. Am J Pathol. 1995;146: 419–28. 7856752 PMC1869839

[16] Deten A, Volz HC, Driest W, Zimmer HG. Cardiac cytokine expression is upregulated in the acute phase after myocardial infarction. Experimental studies in rats. Cardiovasc Res. 2002;55: 329–340. doi: 10.1016/S0008-6363(02)00413-3 12123772 10.1016/s0008-6363(02)00413-3

[17] Kurrelmeyer KM, Michael LH, Baumgarten G, Taffet GE, Peschon JJ, Sivasubramanian N, et al Endogenous tumor necrosis factor protects the adult cardiac myocyte against ischemic-induced apoptosis in a murine model of acute myocardial infarction. Proc Natl Acad Sci U S A. 2000;97: 5456–61. doi: 10.1073/pnas.070036297 10779546 10.1073/pnas.070036297 PMC25850

[18] Maekawa N, Wada H, Kanda T, Niwa T, Yamada Y, Saito K, et al Improved myocardial ischemia/reperfusion injury in mice lacking tumor necrosis factor-alpha. J Am Coll Cardiol. 2002;39: 1229–1235. doi: 10.1016/S0735-1097(02)01738-2 11923051 10.1016/s0735-1097(02)01738-2

[19] Saxena A, Chen W, Su Y, Rai V, Uche OU, Li N, et al IL-1 induces proinflammatory leukocyte infiltration and regulates fibroblast phenotype in the infarcted myocardium. J Immunol. 2013;191: 4838–48. doi: 10.4049/jimmunol.1300725 24078695 10.4049/jimmunol.1300725 PMC3822582

[20] Fischer P, Hilfiker-Kleiner D. Role of gp130-mediated signalling pathways in the heart and its impact on potential therapeutic aspects. Br J Pharmacol. 2008;153 Suppl: S414–27. doi: 10.1038/bjp.2008.1 18246092 10.1038/bjp.2008.1 PMC2268054

[21] Fuchs M, Hilfiker A, Kaminski K, Hilfiker-Kleiner D, Guener Z, Klein G, et al Role of interleukin-6 for LV remodeling and survival after experimental myocardial infarction. FASEB J. 2003;17: 2118–2120. doi: 10.1096/fj.03-0331fje 12958147 10.1096/fj.03-0331fje

[22] Gaffen SL. An overview of IL-17 function and signaling. Cytokine. 2008;43: 402–7. doi: 10.1016/j.cyto.2008.07.017 18701318 10.1016/j.cyto.2008.07.017 PMC2582446

[23] Miossec P, Korn T, Kuchroo VK. Interleukin-17 and type 17 helper T cells. N Engl J Med. 2009;361: 888–98. doi: 10.1056/NEJMra0707449 19710487 10.1056/NEJMra0707449

[24] Liao Y-H, Xia N, Zhou S-F, Tang T-T, Yan X-X, Lv B-J, et al Interleukin-17A contributes to myocardial ischemia/reperfusion injury by regulating cardiomyocyte apoptosis and neutrophil infiltration. J Am Coll Cardiol. 2012;59: 420–9. doi: 10.1016/j.jacc.2011.10.863 22261166 10.1016/j.jacc.2011.10.863 PMC3262985

[25] Cheng X, Yu X, Ding Y-J, Fu Q-Q, Xie J-J, Tang T-T, et al The Th17/Treg imbalance in patients with acute coronary syndrome. Clin Immunol. 2008;127: 89–97. doi: 10.1016/j.clim.2008.01.009 18294918 10.1016/j.clim.2008.01.009

[26] Simon T, Taleb S, Danchin N, Laurans L, Rousseau B, Cattan S, et al Circulating levels of interleukin-17 and cardiovascular outcomes in patients with acute myocardial infarction. Eur Heart J. 2013;34: 570–7. doi: 10.1093/eurheartj/ehs263 22956509 10.1093/eurheartj/ehs263

[27] Thygesen K, Alpert JS, Jaffe AS, Simoons ML, Chaitman BR, White HD, et al Third universal definition of myocardial infarction. Eur Heart J. 2012;33: 2551–67. doi: 10.1093/eurheartj/ehs184 22922414 10.1093/eurheartj/ehs184

[28] Al-Saadany HM, Hussein MS, Gaber RA, Zaytoun HA. Th-17 cells and serum IL-17 in rheumatoid arthritis patients: Correlation with disease activity and severity. Egypt Rheumatol. 2016;38: 1–7. doi: 10.1016/j.ejr.2015.01.001

[29] de Oliveira PSS, Cardoso PRG, de A Lima EV, Pereira MC, Duarte ALBP, da R Pitta I, et al IL-17A, IL-22, IL-6, and IL-21 Serum Levels in Plaque-Type Psoriasis in Brazilian Patients. Mediators Inflamm. Hindawi; 2015;2015: 819149 doi: 10.1155/2015/819149 26351408 10.1155/2015/819149 PMC4550763

[30] Pavlovic V, Dimic A, Milenkovic S, Krtinic D. Serum levels of IL-17, IL-4, and INFγ in Serbian patients with early rheumatoid arthritis. J Res Med Sci. Medknow Publications; 2014;19: 18–22. Available: http://www.ncbi.nlm.nih.gov/pubmed/24672560 PMC3963318 24672560

[31] Ndongo-Thiam N, Miossec P. A cell-based bioassay for circulating bioactive IL-17: application to destruction in rheumatoid arthritis. Ann Rheum Dis. 2015;74: 1629–1631. doi: 10.1136/annrheumdis-2014-207110 25902792 10.1136/annrheumdis-2014-207110

[32] Mewton N, Thibault H, Roubille F, Lairez O, Rioufol G, Sportouch C, et al Postconditioning attenuates no-reflow in STEMI patients. Basic Res Cardiol. 2013;108: 383 doi: 10.1007/s00395-013-0383-8 24022373 10.1007/s00395-013-0383-8

[33] Suinesiaputra A, Bluemke DA, Cowan BR, Friedrich MG, Kramer CM, Kwong R, et al Quantification of LV function and mass by cardiovascular magnetic resonance: multi-center variability and consensus contours. J Cardiovasc Magn Reson. 2015;17: 63 doi: 10.1186/s12968-015-0170-9 26215273 10.1186/s12968-015-0170-9 PMC4517503

[34] Viallon M, Jacquier A, Rotaru C, Delattre BMA, Mewton N, Vincent F, et al Head-to-head comparison of eight late gadolinium-enhanced cardiac MR (LGE CMR) sequences at 1.5 tesla: from bench to bedside. J Magn Reson Imaging. 2011;34: 1374–87. doi: 10.1002/jmri.22783 21972032 10.1002/jmri.22783

[35] Hashmi S, Zeng QT. Role of interleukin-17 and interleukin-17-induced cytokines interleukin-6 and interleukin-8 in unstable coronary artery disease. Coron Artery Dis. 2006;17: 699–706. doi: 10.1097/01.mca.0000236288.94553.b4 17119379 10.1097/01.mca.0000236288.94553.b4

[36] Liebetrau C, Nef HM, Dörr O, Gaede L, Hoffmann J, Hahnel A, et al Release kinetics of early ischaemic biomarkers in a clinical model of acute myocardial infarction. Hear. 2014;100: 652–657. doi: 10.1136/heartjnl-2013-305253 24488609 10.1136/heartjnl-2013-305253

[37] Eid RE, Rao D a, Zhou J, Lo SL, Ranjbaran H, Gallo A, et al Interleukin-17 and interferon-gamma are produced concomitantly by human coronary artery-infiltrating T cells and act synergistically on vascular smooth muscle cells. Circulation. 2009;119: 1424–32. doi: 10.1161/CIRCULATIONAHA.108.827618 19255340 10.1161/CIRCULATIONAHA.108.827618 PMC2898514

[38] Metawi SA, Abbas D, Kamal MM, Ibrahim MK. Serum and synovial fluid levels of interleukin-17 in correlation with disease activity in patients with RA. Clin Rheumatol. Springer-Verlag; 2011;30: 1201–1207. doi: 10.1007/s10067-011-1737-y 21874405 10.1007/s10067-011-1737-y

[39] Oppenheim JJ, Zachariae COC, Mukaida N, Matsushima K. Properties of the novel proinflammatory supergene “intercrine” cytokine family. Annu Rev Immunol. 1991;9: 617–648. doi: 10.1146/annurev.iy.09.040191.003153 1910690 10.1146/annurev.iy.09.040191.003153

[40] Wernette Hammond ME, Lapointe GR, Feucht PH, Hilt S, Gallegos CA, Gordon CA, et al IL-8 induces neutrophil chemotaxis predominantly via type I IL-8 receptors. J Immunol. 1995;155: 1428–33. 7636208

[41] Knorr M, Münzel T, Wenzel P. Interplay of NK cells and monocytes in vascular inflammation and myocardial infarction. Frontiers in Physiology. 2014 doi: 10.3389/fphys.2014.00295 25177297 10.3389/fphys.2014.00295 PMC4132269

[42] Gabriel AS, Martinsson A, Wretlind B, Ahnve S. IL-6 levels in acute and post myocardial infarction: their relation to CRP levels, infarction size, left ventricular systolic function, and heart failure. Eur J Intern Med. 2004;15: 523–528. doi: 10.1016/j.ejim.2004.07.013 15668089 10.1016/j.ejim.2004.07.013

[43] Ritschel VN, Seljeflot I, Arnesen H, Halvorsen S, Weiss T, Eritsland J, et al IL-6 signalling in patients with acute ST-elevation myocardial infarction. Results Immunol. 2014;4: 8–13. doi: 10.1016/j.rinim.2013.11.002 24707455 10.1016/j.rinim.2013.11.002 PMC3973821

[44] Ørn S, Manhenke C, Ueland T, Damås JK, Mollnes TE, Edvardsen T, et al C-reactive protein, infarct size, microvascular obstruction, and left-ventricular remodelling following acute myocardial infarction. Eur Heart J. 2009;30: 1180–6. doi: 10.1093/eurheartj/ehp070 19299430 10.1093/eurheartj/ehp070

[45] Chia S, Nagurney JT, Brown DFM, Raffel OC, Bamberg F, Senatore F, et al Association of leukocyte and neutrophil counts with infarct size, left ventricular function and outcomes after percutaneous coronary intervention for ST-elevation myocardial infarction. Am J Cardiol. Elsevier Inc.; 2009;103: 333–7. doi: 10.1016/j.amjcard.2008.09.085 19166685 10.1016/j.amjcard.2008.09.085

[46] Deftereos S, Giannopoulos G, Angelidis C, Alexopoulos N, Filippatos G, Papoutsidakis N, et al Anti-inflammatory treatment with colchicine in acute myocardial infarction: A pilot study. Circulation. 2015;132: 1395–1403. doi: 10.1161/CIRCULATIONAHA.115.017611 26265659 10.1161/CIRCULATIONAHA.115.017611

